# Direct electric stimulation to increase cerebrovascular function

**DOI:** 10.3389/fnsys.2015.00054

**Published:** 2015-03-30

**Authors:** Victor M. Pulgar

**Affiliations:** ^1^Biomedical Research and Infrastructure Center, Faculty of Natural and Physical Sciences, Winston-Salem State UniversityWinston-Salem, NC, USA; ^2^Hypertension and Vascular Research Center, Wake Forest School of MedicineWinston-Salem, NC, USA; ^3^Department of Obstetrics and Gynecology, Wake Forest School of MedicineWinston-Salem, NC, USA

**Keywords:** brain Stimulation, cerebral blood flow (CBF), neurovascular coupling, functional hyperemia, transcranial direct current stimulation (tDCS)

Various conditions affecting the cerebral vasculature may lead to cumulative damage and thus deterioration of brain function, in what has been called vascular cognitive impairment (Gorelick et al., 2011). Consequently, it makes sense that an increase in glucose and oxygen produced by an increase in blood flow may augment brain function. Since its rediscovery some years ago transcranial direct electric stimulation (tDCS) has attracted interest as potential therapy for patients with neurological impairments. This opinion article aims to succinctly review the mechanisms involved in neurogenic control of the cerebral blood flow (CBF) highlighting the potential of direct electrical stimulation targeting cerebral micro vessels to enhance brain function.

With the highest oxygen consumption than any other organ in the body, the brain utilizes around 20% of the total resting oxygen, making it an organ highly dependent on blood supply (Hossmann, 1994). Moreover, a direct relationship between the development of neurodegenerative diseases and impairment of CBF has been postulated (Farkas and Luiten, 2001).

The appropriate delivery of nutrients and oxygen to the brain tissue is regulated by mechanisms including cerebral autoregulation, vascular reactivity and neurovascular coupling. The autoregulatory properties of cerebral circulation make CBF independent of systemic blood pressure. Therefore, over a physiological range of pressure cerebral arteries relax when systemic pressure decreases and constrict when systemic pressure increases (Heistad and Kontos, 1983). Similarly, reactivity of the brain blood vessels to pH and CO_2_ has been suggested to link neuronal metabolic changes to cerebral blood flow.

## The neurovascular unit

One of the unique characteristics of the brain circulation is the intimate contact between blood vessels, neurons, and glia. Thus, neurons, glia, and vascular cells are structurally and functionally related in what is called the “neurovascular unit” (Iadecola, 2004). Since brain *P*O_2_ is tightly regulated in relation to local brain activity, the neurovascular unit provides a framework for the functional interactions responsible for this concerted regulation. Thereby, functional hyperemia means that blood flow will increase in brain areas with increased activity.

From an anatomical point of view, pial arteries traveling on the surface of the brain are highly innervated with terminals coming from the peripheral nervous system (extrinsic innervation) (Hamel, 2006). These vessels are surrounded by the Virchow-Robin's space which gradually disappears as vessels enter the brain parenchyma. Cerebral arteries entering the brain parenchyma lose extrinsic innervation and come into intimate contact with neuronal and glial cells (intrinsic innervation) (Iadecola, 2004). Functions of these two vascular compartments, macro and micro vessels, involve regulation of global blood supply, as wells as control of local CBF and brain blood barrier permeability respectively.

It is currently accepted that postsynaptic increases in [Ca^++^]_i_ due to activation of glutamate receptors during synaptic transmission activate the production of vasoactive mediators.

Several mediators such as neurotransmitters, adenosine, arachidonic acid metabolites, nitric oxide (NO), hydrogen and potassium, have been suggested to mediate increases in CBF (Iadecola, 2004).

Due to their close contact with blood vessels, astrocytes are suggested to play an important role in functional hyperemia. Astrocyte's end-feet surround brain micro capillaries, and may mediate neuron-blood vessel communication and thus neuronal activity-induced blood flow changes (Zonta et al., 2003). Vasodilatory as well as vasoconstrictor activities have been ascribed to glial cells (Metea and Newman, 2006), with an important role for glial eNOS in mediating vasodilatation (Stobart et al., 2013). New evidences also point to astrocytes as relevant components of the recently described “glymphatic pathway,” an important mechanism for clearance of solutes from the brain (Iliff et al., 2012). Thus, aquaporin water channels in astrocyte's end feet would couple paravascular pathways for the vectorial convective flow of waste products from arterial toward venous routes, with solutes ultimately clearing the brain through the lymphatic system (Nedergaard, 2013).

The brain endothelium is a highly specialized tissue mediating several physiological functions, such as thrombosis, adhesion, permeability and angiogenesis (Daneman and Prat, 2015). A protective function against cerebral dysfunction has been proposed for the brain vessel's endothelium consistent with the predominant role of endothelial dysfunction in several cerebrovascular diseases. Importantly, *in vivo* experiments have shown that endothelial cell-derived NO mediates cortical hyperemia induced by basal forebrain electrical stimulation (Zhang et al., 1995).

Pericytes, cells located outside of the microvessels in intimate contact with endothelium and astrocyte end-feet, are more frequent on microvessels of the retina and brain and thought to regulate blood flow (Kutcher and Herman, 2009). Pericytes are considered important components of the neurovascular unit as regulators of the brain blood barrier function and also potential mediators of brain vascular dysfunction (Hamilton et al., 2010). Among the properties identified include contraction, hemostasis and angiogenesis. Given their contractile properties, pericytes may act as surrogates of smooth muscle cells in brain microvessels.

Dysfunctional interactions within the neurovascular unit have the potential to lead to brain pathophysiological alterations. Impaired endothelial cell-astrocytes or endothelial cell-pericytes signaling may cause brain blood barrier disruption (Zlokovic, 2008), whereas altered coupling between neuronal activity and vascular responses may contribute to spreading depression (Dreier, 2011).

## tDCS and brain perfusion

Effects of electrical stimulation on the brain have been known for centuries (Priori, 2003). Work in the rat primary motor region showed that electrical stimulation may increase, decrease, or silence neuron's firing (Bindman et al., 1964; Purpura and McMurtry, 1965). These animal studies showed that anodal stimulation caused depolarization, whereas cathodal stimulation caused hyperpolarization, thus increasing the probability for a neuron to produce an action potential. tDCS has been rediscovered as a non-invasive promising tool to modulate brain activity and as a potential treatment for psychiatric and neurological disorders (Priori, 2003; Filmer et al., 2014). An increasing number of studies have reported that tDCS modulates synaptic transmission by regulating levels of neurotransmitters such as GABA, glutamate, serotonin, and dopamine, among others (Nitsche et al., 2008).

Reports showing that stimulation of cerebellar neurons increased diameter of both adjacent arterioles and the upstream vessels, provided a demonstration of the propagation of vascular responses induced by increased neural activity (Iadecola et al., 1997). Importantly, cerebellar stimulation at the fastigial nucleus, reduced ischemia induced by medial cerebral artery occlusion in rats through NO-mediated hemodynamic mechanisms (Zhang and Iadecola, 1993).

**Figure 1:**
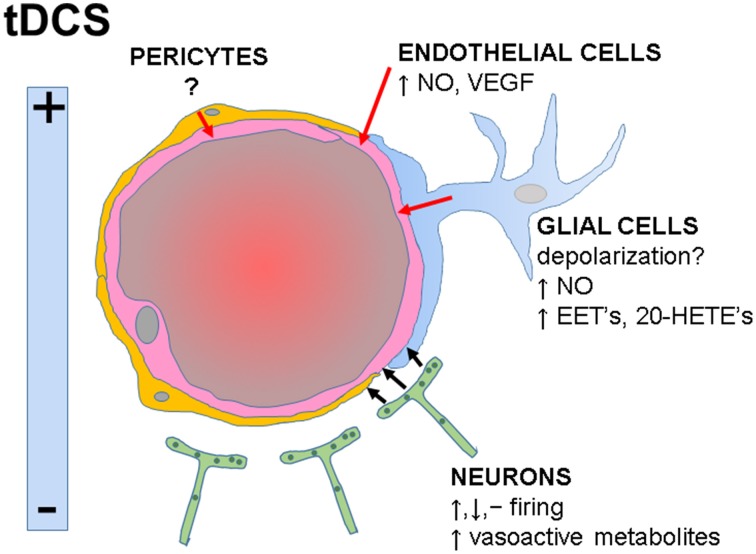
**Functional interactions within the neurovascular unit and potential influences of tDCS on specific cell types**. tDCS-induced electrical fields may increase, decrease or have no effects on neuronal firing rates. Neuronal-derived metabolites directly or indirectly activate endothelial cells (black arrows), inducing vasodilatation. Potential direct effects of tDCS on glial, endothelial cells, and pericytes are indicated (red arrows). NO, nitric oxide; VEGF, vascular endothelial growth factor; EET's, epoxyeicosatrienoic acids; 20-HETE's, 20-hydroxyeicosatetraeonic acids.

tDCS in humans is performed by applying direct current over the scalp using electrodes and its effects depend on the size, polarity and position of the electrodes, current intensity, duration of stimulation, and tissue properties (DaSilva et al., 2011). Given the intimate relationship between neuronal activity and CBF, it is expected that tDCS will increase brain perfusion, as shown in animal (Han et al., 2014) and human (Zheng et al., 2011) studies. The opinion presented in this article is that in addition to the changes in neuronal-derived metabolites, evidences showing responses to electrical stimulation in non-neuronal cells suggest that tDCS acting on these cells has also the potential to modulate brain perfusion (Figure 1). Thus, understanding the vascular effects of tDCS may improve the treatment of diseases associated with vascular dysfunction.

Direct effects of electric stimulation on neurons *in vitro* include alignment of neurites perpendicular to the electric field, increased growth and migration (Pan and Borgens, 2012). In mouse coronal slices, a role for electrical stimulation-induced synaptic plasticity was demonstrated, an effect that may underlie implications of tDCS on motor learning (Fritsch et al., 2010). Results obtained in rat brain slices suggested that electrical stimulation modulates long term potentiation in a polarity-specific manner supporting a regulatory role of tDCS on synaptic plasticity (Ranieri et al., 2012).

Described effects of electrical stimulation on astrocytes *in vitro* include changes in metabolism depending on field polarization and applied voltage (Huang et al., 1997), as well as migration and perpendicular alignment (Pelletier et al., 2014). A theoretical analysis concluded that tDCS has the potential to directly stimulate glial cells since the tDCS-induced changes in membrane potential are similar to the changes induced in astrocytes during neuronal activation (Ruohonen and Karhu, 2012).

The effects of electric stimulation on endothelial cells *in vitro* include the alignment perpendicular to the direction of the electrical field, migration, and elongation (Zhao et al., 2012). These effects are associated with increases in VEGF production, suggesting that electrical stimulation may modulate angiogenesis (Zhao et al., 2012). Conversely, the data from brain slices, including effects on synaptic plasticity (Fritsch et al., 2010) are obtained in the absence of circulation, which may indicate that there is no endothelial contribution to the neuronal effects of electric stimulation. However, endothelial cells in culture exposed to a low physiological electrical field (3.3 mV/mm) showed increased NO production (Trivedi et al., 2013), suggesting a direct route by which electric stimulation may increase brain perfusion. Modeling of the electric properties of the brain suggests that the electric field generated during tDCS in humans is around 1 mV/mm (Neuling et al., 2012) indicating that endothelial cell-dependent responses may be triggered during tDCS.

The proposed role of pericytes in the neurovascular unit suggests that pericytes may transduce signals from neurons to endothelial cells (Hall et al., 2014). Thus, during neuronal activation glutamate release produces prostaglandin E_2_ which in turn will induce capillaries vasodilatation by activating K^+^ currents in pericytes. These are excitable cells and a direct effect of tDCS on pericyte's membrane potential may hyperpolarize it and induce vasodilatory signals. Whether pericyte-mediated responses to tDCS are playing a role in the tDCS effects remains to be elucidated.

Although general agreement has been observed between animal and human studies (Bennabi et al., 2014), it is necessary to note that stimulating parameters used in animal *in vivo* and in *in vitro* protocols are higher than those used in humans where a maximum current density of ~0.28 A/m^2^ is used (Im et al., 2012). In contrast a maximum safe stimulation in rats was reported at 142.9 A/m^2^ (Liebetanz et al., 2009).

## tDCS and augmentation of brain function

In humans, evidences indicate the potential of tDCS to increase cognitive, motor and memory function. For example, tDCS may enhance gesture comprehension by improving gesture and language integration (Cohen-Maximov et al., 2014), a result especially relevant in cases of autism where patients have difficulties processing symbolic gestures (Baron-Cohen, 1988). Anodal tDCS administered repeatedly facilitates language (Meinzer et al., 2014) and motor skill learning (Zimerman et al., 2013). tDCS has also been shown to produce long-lasting effects on number processing (Cohen Kadosh et al., 2010) and there is increasing interest in the applicability of tDCS for memory enhancement (Bennabi et al., 2014).

However, not all tDCS studies have observed positive effects. Thus, the rate of motor sequence learning is increased by anodal tDCS and decreased by cathodal stimulation, whereas tDCS applied prior to the motor task slowed learning (Stagg et al., 2011). Also cerebellar tDCS has been shown to impair practice-dependent improvement in a working memory task (Ferrucci et al., 2008), whereas tDCS applied to pre-frontal cortex disrupts sensory-motor training (Filmer et al., 2013). Clearly, the research describing the efficacy of tDCS for motor and cognitive improvement is still inconclusive.

Brain blood flow responses to specific tasks may involve cortical and subcortical structures, as seen in the attention-derived effects on flow in both the visual cortex and the lateral geniculate nucleus (O'Connor et al., 2002). Thus, it is relevant to point out that tDCS may also modulate blood flow in subcortical structures (Lang et al., 2005; Nonnekes et al., 2014) demonstrating broader effects of tDCS on CBF.

Recent technical developments applying tDCS simultaneously with electroencephalography and brain blood flow measurements (Dutta et al., 2015) will help to integrate tDCS with modulation of vascular function and ultimately changes in human behavior.

## Perspectives and future directions

In order to expand direct therapeutic applicability, tDCS needs to overcome the challenges related to inter- and intra-subject variability and parameters of stimulation impacting neuroplasticity (i.e., short *vs*. long term stimulation), among others. Simultaneous determination of vascular signals and cognitive performance during tDCS will help to integrate electrical stimulation with vascular functioning and changes in behavior. The relationships between neuronal and vascular effects are complex and it is proven difficult to differentiate between the effects of electrical stimulation on those two tissues, it is however conceivable that in addition to the vascular effects of neuronal-derived metabolites, direct effects of tDCS on non-neuronal cells especially glial and endothelial cells modulate brain perfusion. Thus, a deeper understanding of the effects tDCS have on non-neuronal members of the neurovascular unit is essential.

### Conflict of interest statement

The authors declare that the research was conducted in the absence of any commercial or financial relationships that could be construed as a potential conflict of interest.
